# Identification of Diverse Integron and Plasmid Structures Carrying a Novel Carbapenemase Among *Pseudomonas* Species

**DOI:** 10.3389/fmicb.2019.00404

**Published:** 2019-03-04

**Authors:** Eleni Liapis, Maxime Bour, Pauline Triponney, Thomas Jové, Jean-Ralph Zahar, Benoît Valot, Katy Jeannot, Patrick Plésiat

**Affiliations:** ^1^Centre National de Référence de la Résistance aux Antibiotiques, CHRU Jean Minjoz, Besançon, France; ^2^CNRS, UMR 6249 Chrono-Environnement, Besançon, France; ^3^INSERM UMR 1092, Université de Limoges, CHU Limoges, Limoges, France; ^4^Département de Microbiologie Clinique, AP-HP, CHU Avicenne, Bobigny, France

**Keywords:** *Pseudomonas*, IMP carbapenemases, plasmids, integrons, diffusion

## Abstract

A novel carbapenem-hydrolyzing beta-lactamase, called IMP-63, was identified in three clonally distinct strains of *Pseudomonas aeruginosa* and two strains of *Pseudomonas putida* isolated within a 4 year timeframe in three French hospitals. The *bla*_IMP–63_ gene that encodes this carbapenemase turned out to be located in the variable region of four integrons (In*1297*, In*1574*, In*1573*, and In*1572*) and to coexist with novel or rare gene cassettes (*fosM*, *gcu170*, *gcuF1*) and insertion elements (IS*Psp7v*, IS*Pa16v*). All these integrons except one (In*1574*) were flanked by a copy of insertion sequence IS*Pa17* next to the *orf6* putative gene, and were carried by non-conjugative plasmids (pNECK1, pROUSS1, pROUSS2, pROUE1). These plasmids exhibit unique modular structures and partial sequence homologies with plasmids previously identified in various non-fermenting environmental Gram-negative species. Lines of evidence suggest that IS*Pa17* promoted *en bloc* the transposition of IMP-63-encoding integrons on these different plasmids. As demonstrated by genotyping experiments, isolates of *P. aeruginosa* harboring the 28.9-kb plasmid pNECK1 and belonging to international “high-risk” clone ST308 were responsible for an outbreak in one hospital. Collectively, these data provide an insight into the complex and unpredictable routes of diffusion of some resistance determinants, here *bla*_IMP–63_, among *Pseudomonas* species.

## Introduction

The *Pseudomonas putida* group is composed of several closely related species that live in the soil and surface waters as saprophytes within complex communities (Gomila et al., 2015). Though poorly pathogenic for humans these Gram-negative bacteria can occasionally generate acute or chronic infections in fragile patients. Thus species such as *P. putida (sensu stricto)*, *P. monteilii*, and *P. mosselii* are regularly isolated from clinical samples (Yang et al., 1996). Because of the intrinsic susceptibility of these bacteria to many antibiotics and their low virulence, the issue of their resistance to chemotherapy has not been addressed specifically and has not attracted much attention so far. However, the presence of multidrug resistant strains of *P. putida* (here, used as a generic name for several species or subspecies of the *P. putida* complex) in the hospital setting is increasingly reported (Raphael and Riley, 2017). As for the well-known human pathogen *P. aeruginosa*, these species are prone to become resistant to major antibiotic families including aminoglycosides, fluoroquinolones and ß-lactams. More alarming are the observations that some of them produce carbapenem-inactivating ß-lactamases (so-called carbapenemases), and that these enzymes are of the same types as those found in epidemic clones of *P. aeruginosa* (Oliver et al., 2015; Raphael and Riley, 2017). Indeed metallo-ß-lactamases (MBLs) of VIM- and IMP-types are the most prevalent carbapenemases in carbapenem-resistant *P. aeruginosa* and *P. putida*. Their genetic determinants can be borne by the bacterial chromosome or by plasmids (Lee et al., 2002; Docquier et al., 2003; Yomoda et al., 2003; Marchiaro et al., 2010). Given the phylogenetic proximity of fluorescent *Pseudomonas*, plasmid exchanges are expected to occur freely among these species (Boronin, 1992). However, whether some of these bacteria constitute a reservoir of resistance determinants for the others remain poorly understood (Juan et al., 2010). In this study, we show that the diffusion of a rare variant of MBL IMP-12, named IMP-63, among clinical strains of *P. aeruginosa* and *P. putida* occurred through a complex scenario involving several genetic cargos.

## Materials and Methods

### Bacterial Strains and Microbiology Techniques

*Pseudomonas aeruginosa* strains Pae1151 to Pae1156, and Pae1133 were collected between November 2011 and July 2012 during an outbreak that occurred in hospital H1, Paris, France. Strain Pae2567 was isolated in the same hospital 2 years later, in 2014. These isolates were found in the following clinical samples: rectal swabs (*n* = 2), urine (*n* = 2), tracheal aspirate (*n* = 1), catheter (*n* = 1), blood culture (*n* = 1), and eye swab (*n* = 1). A strain of *P. putida* named Pp917 was recovered from the stools of an oncology patient admitted to hospital H2, Paris, in 2012, while a *P. aeruginosa* called Pae3940 was detected in the same unit of this hospital in 2016, in the rectal swab from another patient. *Pseudomonas putida* Pp2223 was responsible for a urinary tract infection in a patient hospitalized in hospital H3 (140 km distant from Paris) in 2014. The bacteria were identified at the species level by matrix-assisted laser desorption ionization-time of flight (MALDI-TOF) mass spectrometry using a Bruker MALDI MS equipment. Rifampicin-resistant *P. aeruginosa* strain PU21 was used in MIC experiments as control and as recipient for plasmid transfers (see below) (Loutit et al., 1968). All the bacterial cultures were performed at 35°C ± 1 on Mueller-Hinton agar medium (MHA, BioRad, Marnes-la-Coquette, France) or in Mueller-Hinton broth (MHB, Becton Dickinson, Microbiology Systems, Cockeysville, MD, United States) containing adjusted concentrations of Mg^2+^ and Ca^2+^. MICs of antibiotics were determined in three independent experiments by the microbroth dilution method with Sensititre^®^ microplates containing specifically designed drug panels (ThermoFisher Scientific, Villebon-sur-Yvette, France). Results were recorded after an incubation of 18 h ± 2 and were interpreted according to ongoing EUCAST breakpoints^1^.

### DNA Amplification and Sequencing Methods

Amplification of gene *bla*_IMP–63_ was obtained with a PCR targeting the subgroup of IMP beta-lactamases. The 50 μL reaction mixture contained 3 μL DNA extract, 0.06 μM forward primer IMP2010-F (5′-GTTTATGTTCATACWTCGTT-3′) and reverse primer IMP2010-R1 (5′-GCCAAGCTTCTAAATTTGC-3′), 10 μL MyTaq Red Mix (Bioline, France), 1.25 U MyTaq Red DNA Polymerase (Bioline), 3 μL DMSO and 27.75 μL distilled water. After an initial denaturation step of 5 min at 94°C, the gene was amplified through 30 cycles each consisting in 1 min at 94°C, 1 min hybridization at 51°C, followed by an extension of 1 min at 72°C. A final extension of 7 min at 72°C ended the reaction. Genes encoding IMP enzymes are frequently located into class I integrons. Complete nucleotide sequence of *bla*_IMP–63_ was obtained by PCR and sequencing on both strands with specific primers Hep58 (5′-TCATGGCTTGTTATGACTGT) and Hep59 (5′-GTAGGGCTTATTATGCAGGC), annealing to 5′-CS and 3′-CS conserved sequences of class I integrons, respectively (White et al., 2000).

The whole DNA content of strains Pae1156, Pae2567, Pp917, Pae3940, PU21(pTROUS1), and Pp2223 was sequenced with the Illumina technology. Briefly, total bacterial DNA was extracted from overnight cultures by using PureLink Genomic DNA Mini Kit (ThermoFisher Scientific), then was quantified with a NanoDrop spectrophotometer (Ozyme, Montigny le Bretonneux, France), and finally sequenced by Microsynth AG (Balgach, Switzerland) in an Illumina NextSeq sequencer with v2 chemistry, using 2 × 150 paired-end reads. DNA libraries were prepared with Nextera XT DNA Library Preparation Kit (Illumina, San Diego, CA, United States). High throughput sequencing yielded from 6039419 to 6690442 reads per strain, with a coverage depth comprised between 80 and 150×. Antimicrobial resistance genes were identified by uploading the resultant reads to ResFinder 3.1 program available at the Center for Genomic Epidemiology server^2^. *De novo* assembly of paired-end fragments, search of open reading frames (ORFs), and gene annotation were performed by using Basic Local Alignment Search Tool BLASTn^3^ and CLC genomics Workbench 10.0.1 software (Qiagen Bioinformatics, Redwood city, CA, United States). Schematic representation of integrons (Figure 2) was drawn with SnapGene^4^. To establish the plasmid pNECK1 backbone, longer contigs were generated from strain Pae1156 by PacBio SMRT sequencing (CD Genomics, Shirley, NY, United States). The IMP-63-encoding plasmids have been deposited in the NCBI database under the accession numbers MK047609 (pNECK1), MK047610 (pTROUS1), MK047611 (pTROUS2), and MK047608 (pROUE1).

### Strain Typing

The sequence types (STs) of *P. aeruginosa* isolates were determined according to the scheme proposed by van Mansfeld et al. (2009). Internal sequences of housekeeping genes *acsA*, *aroE*, *guaA*, *mutL*, *nuoD*, *ppsA*, and *trpE*, as determined by whole genome sequencing or specific PCRs (van Mansfeld et al., 2009), were uploaded to MLST web site^5^ to define STs. In addition, the clonality of the strains involved in hospital H1 outbreak was investigated by MLVA (Multiple-Locus Variable-Number Tandem-Repeat Analysis) targeting microsatellites ms142, ms211, ms212, ms213, ms214, ms215, ms216, ms217, ms222, and ms223 (Vu-Thien et al., 2007). The size of individual amplicons was determined for each strain by agarose gel electrophoresis. The banding patterns of strains Pae1151 to Pae1156 were strictly identical, but different from that of the late isolate Pa2567 (data not shown). When mentioned in the text, strains of *P. aeruginosa* were serotyped by glass agglutination with O-antiserums from BioRad, according to the Habbs scheme.

### Gene Transfer Experiments and Plasmid Characterization

Plasmid transfers between the IMP-63-producing strains and rifampicin-resistant *P. aeruginosa* PU21 were attempted. Briefly, 100 μL of log-phase donor and recipient cultures in MHB were mixed and deposited on a 0.45 μm pore size nitrocellulose membrane at the surface of a MHA plate. After 3 h at 35°C ± 1, the bacteria were resuspended in MHB and then cultured overnight on MHA plates containing 150 μg/mL ticarcillin and 200 μg/mL rifampicin to counterselect PU21 transconjugants. When present, resistant colonies were subsequently streaked onto the same selective medium and submitted to plasmid extraction by the alkaline lysis method of Kieser (1984). The transfer of plasmid pNECK1 was visually confirmed by 0.8% agarose gel electrophoresis and ethidium bromide staining. The four plasmids from *E. coli* 39R861 (also named NCTC 50192) were used as calibrators to estimate plasmid size. Transfer of plasmid pTROUS2 from Pa3940 to PU21 was obtained by electro-transformation according to the protocol of Smith and Iglewski (1989).

## Results

### Outbreak Strains of *P. aeruginosa* Producing IMP-63 in Hospital H1, Paris

**FIGURE 1:**
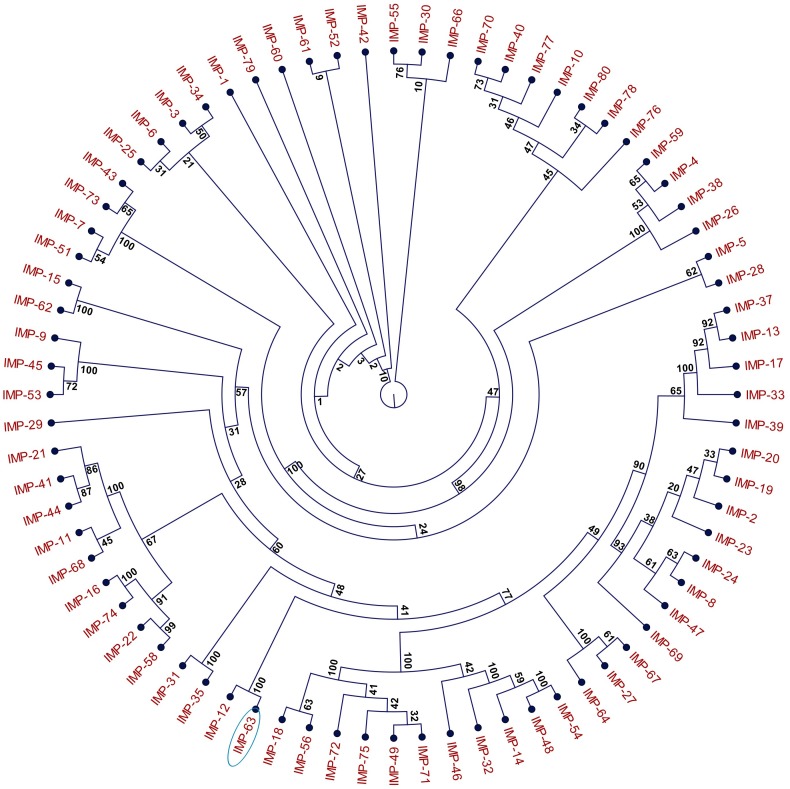
Phylogeny of IMP-type metallo-ß-lactamases (MBLs). The cladogram is based on the amino acid sequences of 76 IMP-type MBLs deposited in the NCBI database (https://www.ncbi.nlm.nih.gov). Construction of the cladogram was performed using the Maximum Likelihood method with 100 replicates. Bootstrap values are indicated next to each branch. In this representation, the length of the branches is irrelevant of evolutionary distance. Enzyme IMP-63 is circled.

Seven multidrug resistant strains of *P. aeruginosa* isolated in hospital H1, Paris, between November 2011 and July 2012 were referred to the French National Reference Center for Antibiotic Resistance (NRC-AR, Besançon) for characterization of their resistance mechanisms to ß-lactams. These outbreak isolates (Pae1133, and from Pae1151 to Pae1156) were found to belong to serotype O:11 by slide agglutination and to display the same drug susceptibility profile. According to the EUCAST breakpoints, they were resistant or intermediate to all the ß-lactams and aminoglycosides tested, but remained susceptible to ciprofloxacin and colistin (see isolate Pae1156 in Table 1). They shared the same genotype ST308 and MLVA profile (data not shown). All of them yielded a positive double-disk synergy test between Zn^2+^ chelator EDTA and imipenem, suggesting the acquisition of a metallo-beta-lactamase (MBL). Use of a PCR targeting known transferable MBL-encoding genes led to the identification of *bla*_IMP–63_, which codes for a new variant of IMP-12. This latter carbapenemase was detected in an Italian strain of *P. putida* in year 2000 (Docquier et al., 2003), and to the best of our knowledge has never been reported since then in the literature. IMP-63 (accession number AOR06148) differs from IMP-12 by a Gly-to-Ser substitution at position 214 and is relatively distant from other IMP-type enzymes, with the closest relatives being IMP-8 and IMP-2 (87.3 and 86.9% sequence identity, respectively). The phylogeny of IMP proteins including IMP-63 is represented Figure 1. The gene *bla*_IMP–63_ sequence was deposited in GenBank under the accession number KX821663.1. Interestingly, additional investigations showed that gene *oprD* which codes for the carbapenem-specific porin OprD in *P. aeruginosa* was disrupted by a new 1,333-bp IS-like element in Pae1156. This finding supports the notion that both enzymatic and membrane impermeability mechanisms contributed to the carbapenem resistance of H1 outbreak strains.

**Table 1:** Drug susceptibility of IMP-63 positive strains of *P. aeruginosa* and *P. putida*.

Strains (origin/plasmid)	MIC (mg/L)											
	TIC	TZP*^a^*	ATM	CAZ	FEP	C/T*^a^*	IPM	MEM	CIP	TM	AKN	CS
Pae1156*^b^* (H1, Paris)	512	32	16	>64	>64	64	64	16	0.25	>32	32	1
Pae2567 (H1, Paris)	32	4	2	32	32	2	2	1	0.5	>32	128	1
Pae3940 (H2, Paris)	128	32	8	64	32	32	2	1	≤0.12	>32	128	1
PU21*^c^*	32	4	4	2	2	1	1	0.5	≤0.12	0.5	4	1
PU21(pNECK1)	256	32	8	>64	64	64	8	4	≤0.12	>32	16	1
PU21(pTROUS1)	256	32	8	>64	>64	64	8	4	≤0.12	>32	64	1
*P. putida*												
Pp917 (H2, Paris)	>512	>256	>128	>64	>64	32	32	>32	>16	>32	64	1
Pp2223 (H3, Rouen)	>512	16	32	>64	64	16	16	32	>16	16	8	1

*^a^With a fixed concentration of tazobactam (4 mg/L). ^b^MIC values were identical for the other Necker outbreak strains Pae1133, Pae1151, Pae1152, Pae1153, Pae1154, Pae1155 (data not presented). ^c^Rifampicin-resistant mutant derived from PAO1. TIC, ticarcillin; TZP, piperacillin/tazobactam; ATM, aztreonam; CAZ, ceftazidime; FEP, cefepime; C/T, ceftolozane/tazobactam; IPM, imipenem; MEM, meropenem; CIP, ciprofloxacin; TM, tobramycin; AKN, amikacin; CS, colistin.*

### Genetic Environment of *bla*_IMP–63_ in *P. aeruginosa* Pae1156

**FIGURE 2:**
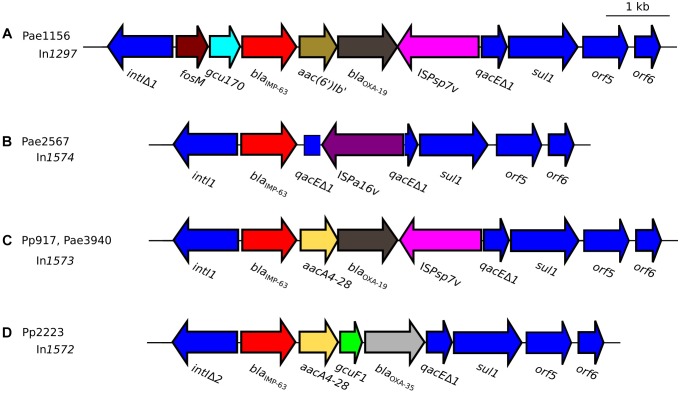
Schematic representation of *bla*_IMP–63_-carrying integrons detected in strains of **(A)** *P. aeruginosa* Pae1156 isolated in Paris, in 2012 during the hospital H1 outbreak; **(B)** *P. aeruginosa* Pae2567 isolated in hospital H1, in 2014; **(C)** *P. putida* Pp917 and *P. aeruginosa* Pae3940 isolated in hospital H2, Paris, in 2012 and 2016, respectively; **(D)** *P. putida* Pp2223 isolated in hospital H3, Rouen, in 2014. Genes that form the conserved backbone of these integrons are represented as deep-blue arrows. Gene *bla*_IMP–63_ is highlighted in red, while the other cassettes present in the variable regions of the integrons are represented with various colors because of their nucleotide sequence diversity.

Most *bla*_IMP_ genes are carried by class I integrons^6^. Whole genome sequencing of strain Pae1156 confirmed the presence of *bla*_IMP–63_ in the variable region of a novel 6-kb integron, dubbed In*1297*. This integron which contains a unique array of gene cassettes and a truncated integrase gene *intI* lacking 110-bp at its 3′ end, is interrupted upstream of canonical *qacEΔ1* and *sul1* genes by a sequence encoding a Thr63Ala, Arg91His, Gly229Ser variant of insertion sequence IS*Psp7* (named IS*Psp7v* in Figure 2). The IS*Psp7* element which belongs to the IS*30* family was described in the environmental *Pseudomonas* sp. strain ZM2 recovered in postflotation tailings samples from Poland (Szuplewska et al., 2014). Immediately downstream of the *intI* truncated sequence (here named *intI*Δ1), *fosM* codes for a new fosfomycin resistance determinant sharing 63% amino acid sequence identity with FosI, and that likely accounts for the high fosfomycin MICs of H1 isolates (>1,024 μg/mL). The function of the second cassette, *gcu170*, remains unknown as those of other *gcu* genes. Located at the third position of the array, *bla*_IMP–63_ is followed by the aminoglycoside-modification gene *aac(6′)-Ib′*, that is predicted to confer a high resistance to gentamicin, tobramycin and netilmicin, and comparatively low resistance to amikacin. Indeed, the Gln101-Ser102-Leu103-Ala104 motif within the active site of AAC(6′)-Ib enzymes results in an amikacin-to-gentamicin switch in resistance, as compared to the Gln/Ser101-Leu102-Leu103-Ala/Ser104 motif (Rather et al., 1992; Casin et al., 2003). Next in the list of In*1297*-borne cassettes, gene *bla*_OXA–19_ is the determinant of an extended-spectrum variant of oxacillinase OXA-35 harboring a Gly167Asp mutation (DBL numbering) (Mugnier et al., 1998). OXA-19 efficiently hydrolyzes newer cephalosporins including ceftazidime, cefepime and ceftolozane (Mugnier et al., 1998). In contrast to *fosM*, *gcu170*, and *bla*_IMP–63_ which, to our knowledge, have never been reported in *P. aeruginosa* before, cassettes *aac(6′)-Ib′* and *bla*_OXA–19_ are rather common in multidrug resistant strains in France (Hocquet et al., 2010).

### Plasmid pNECK1

**FIGURE 3:**
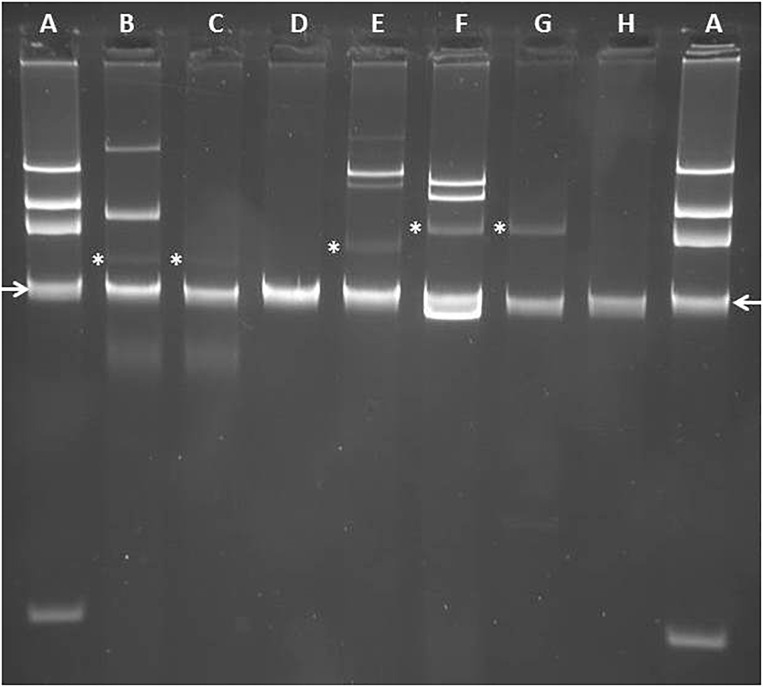
Analysis of plasmid content of IMP-63 positive strains by agarose gel electrophoresis. Plasmids were extracted by using the method of Kieser (1984) and separated by electrophoresis in a 0.8% agarose gel. **(A)** *E. coli* strain 39R861 (NCTC 50192) harboring plasmids of ca 154-, 66-, 38-, and 7-kb, respectively, used as size standards for gel calibration; **(B)** *P. aeruginosa* strain Pae1156 harboring plasmid pNECK1 (marked with an asterisk); **(C)** *P. aeruginosa* strain PU21 transformed with pNECK1; **(D)** *P. aeruginosa* strain Pae2567; **(E)** *P. putida* strain Pp917 harboring plasmid pTROUS2 (marked with an asterisk); **(F)** *P. aeruginosa* strain Pae3940 harboring plasmid pTROUS1 (marked with an asterisk); **(G)** PU21 transformed with pTROUS1; **(H)** *P. putida* strain Pp2223. Location of DNA band corresponding to linear chromosomal fragments is indicated by arrows.

**FIGURE 4:**
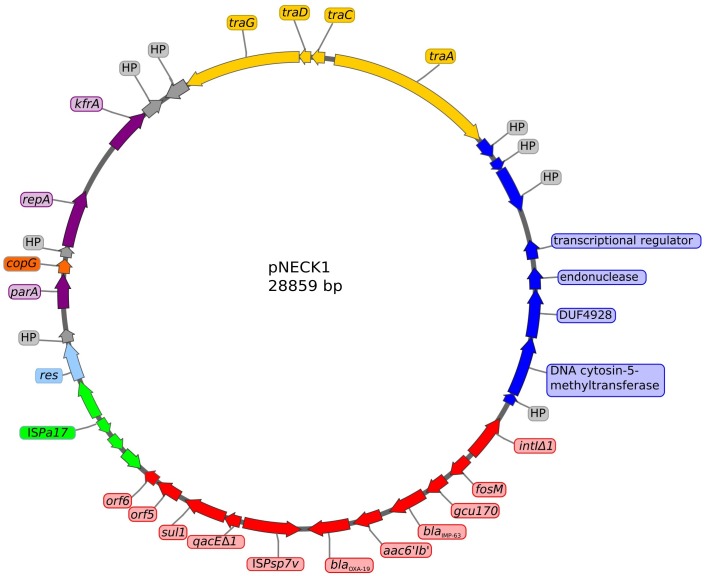
Structure of plasmid pNECK1 harbored by *P. aeruginosa* strain Pae1156. The GC content of this replicon is equal to 63.21%. Arrows in purple color represent genes involved in plasmid housekeeping functions such as partition, replication, and stability. The integron In*1297* structure is indicated in red color, flanked with a copy of insertion sequence IS*Pa17* in green. Genes involved in plasmid transfer are indicated in yellow. Dark-blue arrows correspond to a set of genes previously found in a *Burkholderia cenocepacia* plasmid. Genes of unknown function are shown in gray.

**FIGURE 5:**
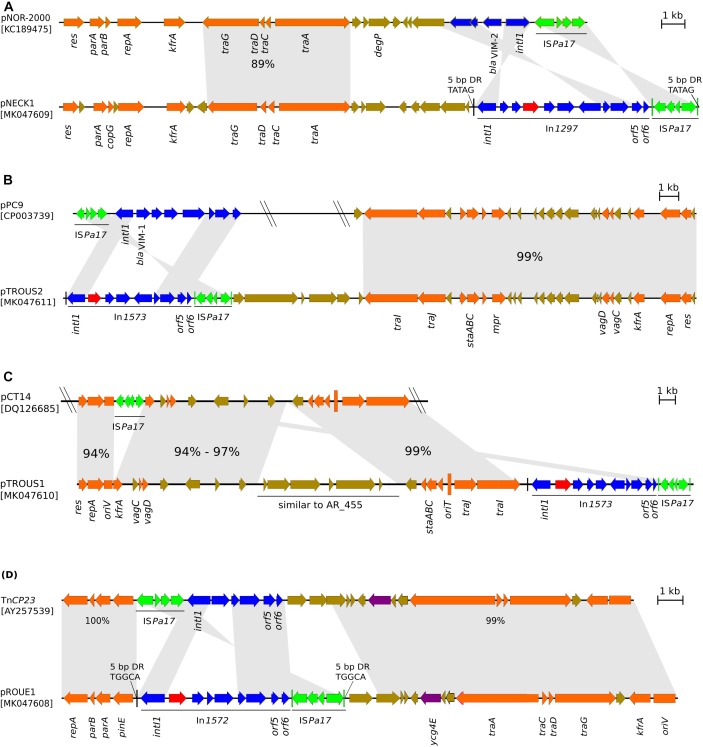
Sequence alignment of the four *bla*_IMP–63_-carrying genetic structures, with structurally closest plasmids. **(A)** Comparison of plasmid pNECK1 with pNOR-2000; **(B)** pTROUS2 with pPC9; **(C)** pTROUS1 with pCT14; **(D)** pROUE1 with Tn*CP23*. Arrows show the direction of transcription. Arrows in orange color represent genes involved in plasmid transfer and housekeeping functions such as replication, partition and stability. DNA regions in plasmid pairs that were found homologous by BLASTn comparison, are connected by gray stretches that take into account gene orientations; the percent nucleotide sequence identity is indicated for relevant homology regions. Genes of integrons are shown in deep-blue color, except cassette *bla*_IMP–63_ highlighted in red. Arrows corresponding to genes of unknown function and IS*Pa17* are colored in brown and in green, respectively. IRR of IS*Pa17* and IRi of Tn*402*-like transposon are mapped and represented by vertical lines in green and black colors, respectively.

Analysis of the plasmid content of strain Pae1156 by agarose gel electrophoresis after extraction by the alkaline lysis method, showed the presence of three bands of ca 30-kb, 60-kb and >200-kb, respectively (Figure 3). Mating-out experiments with rifampicin-resistant *P. aeruginosa* PU21 allowed the transfer at very low frequency (ca 10^−7^ per donor cell) of the 30-kb plasmid that eventually proved to carry the whole In*1297* sequence (see below). The resistance profile of selected transconjugants was similar to that of Pae1156 except for carbapenem MICs, that appeared to be four to eightfold lower for the recipients than for the donor strain [compare PU21(pNECK1) and Pae1156 in Table 1]. These data reinforce the notion that in Pae1156 the activity of carbapenemase IMP-63 is strongly potentiated by the reduced permeability of the outer membrane to carbapenems. The transferred plasmid, dubbed pNECK1, was completely sequenced and assembled in a single contig of 28,859-bp (Figure 4). Its GC content was equal to 63.21%. Search in databases revealed that the replicon is related, though distantly, to several broad-host range IncP plasmids such as pNOR-2000 and pAX22 (Bonnin et al., 2013; Di Pilato et al., 2014). In these plasmids, the defective Tn*402*-like integron platform is flanked at its 5′ end, immediately downstream of integrase gene *intl1*, by a copy of insertion sequence IS*Pa17* and a divergently transcribed resolvase gene (Figure 5). The same module exists in pNECK1 though inserted at the 3′ end of In*1297*, next to a gene of unknown function, *orf6* (Figure 4). As noted elsewhere, IS*Pa17* exhibits an atypical insertion sequence structure as it contains the genes coding for two putative transposases and a type II toxin-antitoxin system. Its presence has been detected on IncP-1 plasmids harbored by various environmental bacterial species (Heuer et al., 2012). IS*Pa17* is bounded by 25-bp inverted repeats (IRL and IRR) whose sequence is close and identical, respectively, to IRi and IRt of Tn*402*-like transposons. While neither ΔTn*402*::In*1297* nor IS*Pa17* is flanked by direct repeats (DRs) in pNECK1, the same 5′-TATAG sequence is adjacent to IRR of IS*Pa17* and to IRi of ΔTn*402*::In*1297* (Figure 5). This sequence signature suggests that both IS*Pa17* and ΔTn*402*::In*1297* have been acquired *en bloc* by plasmid pNECK1 through a single transposition event, possibly driven by the IS*Pa17* transposases. A similar scenario based on the use IRL and IRt sites instead of IRL and IRR by IS*Pa17* transposases has been proposed for acquisition of the IS*Pa17*-ΔTn*402*::In*70* module by plasmid pAX22 (Di Pilato et al., 2014).

Annotation analysis also revealed that most pNECK1 backbone genes involved in fundamental functions such as plasmid partioning (*parA*), replication (*repA*), maintenance (*kfrA*), and transfer (*traA*, *traC*, *traD*, *traG*) have homologs and a similar linear arrangement in various plasmids including pNOR-2000 and pAX22 (Figure 5). However, the closest matches were obtained with DNA contigs from a *Sphingomonas histidinilytica* strain called UM2 (accession number FUYM01000026) and a *Serratia marcescens* strain, AR_0027 (accession number CP0267703). While pNECK1 lacks the *parB* gene present in pNOR-2000 and pAX22, it contains a *copG*-like gene possibly involved in plasmid replication control (Brantl, 2015), as well as several other ORFs of unknown functions.

Interestingly, pNECK1 shares a 5,376-bp fragment with an unnamed plasmid detected in a cystic fibrosis strain of *Burkholderia cenocepacia* isolated in Canada (GenBank accession number CP019671.1) (Lee et al., 2017). Among the 8 ORFs borne by this fragment in pNECK1 (represented as blue arrows in Figure 4), three are predicted to encode a DNA cytosin-5-methyltransferase, a peptide related to AMP-binding superfamily, and a DNA mismatch endonuclease of very short patch repairs, respectively. All these DNA restriction/modification proteins have amino acid sequences from 96 to 99% identical to their counterparts from *B. cenocepacia*. Closely related homologs are also present in *S. histidinilytica* UM2 and *S. marcescens* AR_0027 (data not shown). Their functions are unclear but could potentially insure inter-species propagation of plasmids.

### A Late IMP-63 Positive *P. aeruginosa* Strain in Hospital H1

Isolated in hospital H1 2 years after the outbreak strains, serotype O:11 strain Pae2567 belongs to ST1284 and thereby is clonally different from previous H1 isolates. The bacterium exhibited a high resistance to ceftazidime and cefepime associated with an almost wild-type susceptibility to other ß-lactams including carbapenems (Table 1). The origin of this very unusual susceptibility profile -especially to labile penicillins ticarcillin and piperacillin, remains unclear. However, a double-disk synergy test with EDTA and imipenem confirmed production of a metallo-ß-lactamase by this isolate (Supplemental Figure S1). BLASTn analysis of its total DNA at the NCBI website did not allow the detection of plasmidic elements or IS*Pa17*, a result consistent with the absence of visible plasmid band by agarose gel electrophoresis after cell alkaline lysis (Kieser, 1984; Figure 3). Gene *bla*_IMP–63_ was the unique resistance cassette of a novel class I integron, named In*1574*, that in contrast to In*1297*, contains a full length integrase gene *intl*. The weak promoter variant PcW that was identified within the *intl* coding sequence is known to be associated with a low expression and high rates of excision of resistance gene cassettes (Jové et al., 2010), and could at least partly account for the atypical drug susceptibility pattern described above. Of note, a variant of insertion IS*Pa16* disrupts the *qacEΔ1* sequence in In*1574*. The location of gene *bla*_IMP–63_ in Pae2567 was not investigated further. Its transfer to PU21 could not obtained either by conjugation or electro-transformation.

### IMP-63 Positive *P. aeruginosa* and *P. putida* Strains in Hospital H2, Paris

A multidrug-resistant IMP-63-producing strain of *P. putida*, named Pp917, was identified in another Parisian hospital, H2, in 2012. Its resistance profile to antibiotics is indicated in Table 1. Sequencing of its whole DNA content revealed the presence of a novel integron structure carrying the *bla*_IMP–63_ gene (Figure 2). The cassette array of In*1573* resembles those of In*1297* as it sequentially contains gene cassettes *bla*_IMP–63_, *aacA4-28*, and *bla*_OXA–19_ followed by a divergently oriented copy of IS*Psp7v* (i.e., the same variant as in In*1297*). However, the AAC(6′)-Ib modifying enzyme encoded by *aacA4-28* differs from that determined by the *aac(6′)Ib′* gene of In*1297*, by the presence of Asn83 and Leu102 residues. As mentioned previously, Leu102 provides AAC(6′)-Ib enzymes with a higher catalytic activity on amikacin than on gentamicin as compared with the Ser102 variants (Rather et al., 1992). Also, while In*1297* contains a truncated *intl* gene, this gene has a full-length sequence of 1,014-bp in In*1573*. The plasmid content of strain Pp917 was analyzed by agarose gel electrophoresis. Four plasmids of ca. 35-kb, 100-kb, 150-kb, and >200-kbp, respectively, were detected in this bacterium (Figure 3), but transfer of ticarcillin resistance to recipient *P. aeruginosa* PU21 could not be obtained either by conjugation or by electro-transformation. Integron In*1573* was localized by whole DNA sequencing and multi-alignment experiments on the ca 35-kb plasmid. This plasmid of 34,948-bp and 59.29% GC, named pTROUS2, contains 45 ORFs including backbone genes sharing ≥99% sequence identity with homologs present on a 80,360-bp plasmid (called pPC9) previously found in another *P. putida* strain isolated in the East of France (Molina et al., 2014; Figure 5). Plasmid pPC9 carries an integron encoding the metallo-ß-lactamase VIM-1 and a copy of IS*Pa17* adjacent to the *intl* gene. A copy of IS*Pa17* is also present on pTROUS2 but, as on plasmid pNECK1, this element is located next to *orf6*.

A resistant strain of *P. aeruginosa*, Pae3940, isolated in the same hospital H2 4 years later, in 2016, was also found to produce carbapenemase IMP-63 (Table 1). Its sequence type, ST446, was different from that of the H1 isolates (ST308 and ST1284, respectively). Analysis of its DNA content by high throughput sequencing and subsequent contig assembly revealed the presence of a plasmid of 42,035-bp and 56.43% GC, named pTROUS1. This replicon is composed of 39 ORFs and exhibits similarities with plasmid pCT14 from *Pseudomonas veronii* CT14, a bacterium isolated in the United States from activated sludge (Bramucci et al., 2006; Figure 5). The two replicons share a sequence identity from 94 to 99% over the backbone genes. Plasmid pCT14 which consists of 51 ORFs and is 55,216 nucleotide long, does not harbor integron-like structures but possesses mercury resistance determinants and a copy of IS*Pa17*. More importantly pTROUS1 carries the same integron In*1573* as in plasmid pTROUS2, flanked by a copy of IS*Pa17* next to *orf6*. No DRs adjacent to IRR of IS*Pa17* and to IRi of ΔTn*402*::In*1573* could be identified in pTROUS1 and pTROUS2. It is important to mention that these two plasmids differ in their structures and do not derive from each other (data not shown). While the transfer of pTROUS1 to PU21 could not be obtained by conjugational mating, this was possible by electro-transformation. As indicated in Table 1, the susceptibility profile of transformant PU21(pTROUS1) was fairly similar to that of PU21(pNECK1). In strain Pae3940, pTROUS1 coexists with three other plasmids of ca 18-, 80-, and 100-kb, respectively, whose contribution to antibiotic resistance remains unknown (Figure 3).

### IMP-63 Positive *P. putida* Strain in Hospital H3

With the same methods applied to characterize the other IMP-63-producing strains, a drug resistant strain of *P. putida*, dubbed Pp2223, was identified in 2014 in a third hospital, H3, located 140 km from Paris (Table 1). This bacterium appeared to be genotypically unrelated to *P. putida* Pp917 from hospital H2 when compared by MLST^7^. The novel integron (In*1572*) present in that strain contained a mutation in the *intl* gene resulting in the synthesis of a predicted peptide of 190 amino acids in length instead of the 337 residues of wild-type integrase (Figure 2). This *intl* allele, here called *intlΔ2*, was different from that of integron In*1297*, previously found to encode a 290 amino acid peptide. It should be noted that both mutated forms of the integrase are non-functional since they lack the Tyr312 catalytic site. Analysis of the *aac(6′)Ib* gene located downstream of *bla*_IMP–63_ in In*1572* showed that this cassette, *aacA4-28*, determines the same AAC(6′)-Ib variant (Asn83, Leu102) as that of In*1573* from Pp917. The next gene present in the array, *gcuF1*, was initially identified upstream of gene *bla*_OXA–28_ in the variable region of an integron, In*442*, carried by a *P. aeruginosa* strain from France (Hocquet et al., 2012). Interestingly, extended-spectrum ß-lactamase OXA-28 is a single point variant (Trp164Gly, DBL numbering) of OXA-35, an enzyme able to hydrolyze penicillins, aztreonam and cefepime (Aubert et al., 2001; Poirel et al., 2001). Since transcription of *bla*_OXA–28_ is negatively influenced by cassette *gcuF1* in In*442*, the same may potentially apply to *bla*_OXA–35_ in In*1572* (Hocquet et al., 2012). The occurrence of *gcuF1* in class I integrons is quite rare, which makes this determinant a good marker for epidemiological studies when present in clinical strains (see text footnote 6). The products of *gcuF1* (strain Pp917) and *gcu170* (strain Pae1156) have little in common at the sequence level. Alignment of the DNA reads obtained by high throughput sequencing with sequences from the NCBI database allowed us to gain insight into the genetic support and environment of Pp2223 integron. This element is located on a Tn*CP23*-like structure of 25,062-bp and 61.41% GC, distinct from pNECK1 (Figure 5). The backbone genes of this fragment (32 ORFs in total), that was named pROUE1, display sequence identities of 99–100% with their Tn*CP23* homologs. In some clone C *P. aeruginosa*, plasmid Tn*CP23* is embedded in an integrative conjugative element (ICE) called pKLC102, itself inserted in the bacterial chromosome, in a tRNA_Lys_ gene (Klockgether et al., 2004). However, no pKLC102 specific genes were detected in Pp2223. Given that no plasmid band was visible by agarose gel electrophoresis (Figure 3), the cellular location of pROUE1 remains uncertain but is most likely chromosomal. Attempts to fill the gap between the 5′- and 3′ends of the pROUE1 fragment by long range PCRs failed. Furthermore, transformation and bacterial conjugation experiments even in the presence of helper plasmid pRK2013 (Ditta et al., 1981) were unsuccessful. Like pNECK1, pTROUS1, and pTROUS2 (and unlike Tn*CP23*, pNOR-2000 and pAX22), plasmid pROUE1 carries a copy of insertion sequence IS*Pa17* next to *orf6*, at the 3′ terminus of integron In*1572* (Figure 5). The observation that IRR of IS*Pa17* and IRi of this Tn*402*-like integron are flanked by a same 5′-TGCCA sequence, reinforces the notion that both IS*Pa17* and the ΔTn*402*::In*1572* element have transposed simultaneously on pROUE1.

## Discussion

Since the first description of carbapenemase IMP-1 in a *Serratia marcescens* strain from Japan in 1991 (Osano et al., 1994), reports on the characterization of novel IMP-type ß-lactamases in various bacterial species including *P. aeruginosa* have multiplied worldwide. Despite rather similar Zinc-dependent enzymatic activities, these proteins form a heterogeneous group at the sequence level (as represented by Figure 1). However, some of the IMP-types deposited in the NCBI database (see text footnote 3) differ by limited sequence variations and thus are clearly related, as it is the case of IMP-63 and IMP-12.

Sequential acquisition of resistance determinants by epidemiologically successful clones (also called “high-risk clones”) of *P. aeruginosa* represents a serious public health problem, leaving clinicians with very few therapeutic options to fight nosocomial infections (Del Barrio-Tofino et al., 2017.) By impairing the activity of most antipseudomonal ß-lactams, IMP-type carbapenemases contribute to the evolution of these widespread clones toward pan-drug resistance (Oliver et al., 2015). In this study, the novel metallo-ß-lactamase IMP-63 was identified in one of these clones, involved in an outbreak at H1 hospital, Paris. The detection of other metallo-ß-lactamases such as IMP-1, IMP-8, IMP-13, IMP-45, and VIM-2 in ST308 strains isolated in Japan, Germany, France, China, and India, respectively, confirms the capacity of this international clone to locally collect carbapenemase genes from still unknown environmental reservoirs (reviewed in Oliver et al., 2015). *Pseudomonas putida* may constitute an epidemiological link between such natural reservoirs and *P. aeruginosa* as some hospital strains belonging to the *P. putida* complex were shown to express IMP-1 or IMP-2 in Japan, a country of high endemicity for these ß-lactamases and their variants (Shibata et al., 2003; Yomoda et al., 2003). Simultaneous emergence of IMP-15-positive clinical strains of *P. aeruginosa* and *P. putida* was also noticed in Spain (Gilarranz et al., 2013), while co-occurrence of *P. aeruginosa* and *P. putida* harboring enzyme IMP-19 was recently demonstrated in a French hospital (Amoureux et al., 2017). Of course, other non-fermenting Gram-negative bacilli may take part in the complex diffusion of IMP and VIM determinants (Yan et al., 2001; Amoureux et al., 2017).

In this study, the *bla*_IMP–63_ gene cassette was detected in the variable region of four novel integrons, namely In*1297*, In*1574*, In*1573*, and In*1572* (Figure 2). These integrons were borne by different genetic structures (Figure 5) including four distinct plasmids (pNECK1, pTROUS2, pTROUS1, pROUE1), with In*1573* occurring on two plasmids (pTROUS2, pTROUS1) in *P. aeruginosa* and *P. putida*, respectively. Except the isolates of hospital H1 outbreak, the IMP-63-producing strains of *P. aeruginosa* were clonally unrelated. Several lines of evidence suggest that the location of integron In*1574* in *P. aeruginosa* strain Pae2567 is chromosomal. All the IMP-63-encoding integrons except In*1574* turned out to share the same distinctive feature of being flanked near the putative *orf6* gene by a copy of IS*Pa17*, while this element is located next to the integrase gene *intl* in other ΔTn*402*-like transposon platforms, such as those carried by plasmids pNOR-2000 (Bonnin et al., 2013), pAX22 (Di Pilato et al., 2014), pPC9 (Molina et al., 2014), Tn*CP23* (Klockgether et al., 2004), and pJB12 (Botelho et al., 2017; Figure 5). This observation supports the idea that In*1297*, In*1573* and In*1572* derive from a common *bla*_IMP–63_-carrying transposon whose propagation among *Pseudomonas* strains occurred through complex genetic transfers, involving IS*Pa17*-mediated transposition events and several plasmid carriers. The presence of DR signatures adjacent to IRR of IS*Pa17* and IRi of ΔTn*402*::In*1297* (plasmid pNECK1) or ΔTn*402*::In*1572* (plasmid pROUE1), indicates that IS*Pa17* likely played a major role in the diffusion of *bla*_IMP–63_ on different supports.

With its unique *bla*_IMP–63_ cassette, integron In*1574* might represent the index platform from which the evolution occurred. While displaying different variable regions, the other IMP-63-encoding integrons contain the same sequential arrangement of three gene cassettes, namely (i) *bla*_IMP–63_, (ii) *aac(6′)Ib′* or *aacA4-28*, and (iii) *bla_OXA–19_* or *bla_OXA–35_*. Genes *aac(6′)Ib′* and *aacA4-28* code for closely related aminoglycoside modifying enzymes, while extended-spectrum ß-lactamases (ESBLs) OXA-19 and OXA-35 only differ by a single amino acid residue. Thus is it tempting to speculate that the triad *bla*_IMP–63_-*aacA4*-*bla_OXA_* was present in In*1297*, In*1573*, and In*1572* prior to integration of other elements such as *fosM*, *gcu170*, *gcuF1*, and IS*Psp7v*. The presence of a divergently oriented copy of IS*Psp7v* in the variable region of two of these integrons also strongly suggests that In*1297* evolved from In*1573*, both by acquisition of *fosM* and *gcu170*, and by mutations in gene *aac(6′)Ib′.* Finally, it should be noted that the integron found to carry gene *bla*_IMP–12_, the closest homolog of *bla*_IMP–63_, in an Italian strain of *P. putida* only contained the gene *aacA4* in addition to as second resistance cassette, and that its genetic support (plasmid pVA758) was actually different from the IMP-63-encoding plasmids described here. Given that *bla*_IMP–63_ is a single nucleotide variant of *bla*_IMP–12_, one cannot rule out the possibility that some of the *bla*_IMP–63_ genes detected in France have evolved independently from undetected *bla*_IMP–12_ precursors, thus giving rise to various evolutionary and diffusion events.

In conclusion, this work illustrates how resistance genes can use very complex and unpredictable routes to diffuse among related species such as those belonging to the fluorescent *Pseudomonas* group, within a relatively short timeframe (2011–2016). Acquisition of gene *bla*_IMP–63_ with other resistance determinants, in particular ESBL-encoding genes (e.g., *bla*_OXA–19_ or *bla*_OXA–35_) conferred a multidrug resistance phenotype to several hospital strains of *P. aeruginosa* and *P. putida*.

## Data Availability

The datasets generated for this study can be found in NCBI, MK047608, MK047609, MK047610, MK047611, and KX821663.1.

## Ethics Statement

The clinical strains described in the present study were isolated by public hospital laboratories (H1, H2, H3), accredited by the French Ministry of Health, as part as their routine diagnostic activities. These strains were then transmitted to the NRC-AR (Besançon) for further investigations, accompanied with fully anonymized data. The analyses performed by the NRC did not require additional clinical samples or medical intervention on patients; therefore no patient consent or specific agreement by an ethics committee was needed.

## Author Contributions

PP and KJ contributed to the conception of the study. MB, EL, PT, TJ, and BV performed the data analyses. PP wrote the manuscript. KJ and J-RZ helped to perform the analysis with constructive discussions.

## Conflict of Interest Statement

The authors declare that the research was conducted in the absence of any commercial or financial relationships that could be construed as a potential conflict of interest.

## Footnotes

1 http://www.eucast.org

2 http://www.genomicepidemiology.org

3 https://www.ncbi.nlm.nih.gov

4 http://www.snapgene.com

5 http://pubmlst.org/paeruginosa/

6 http://integrall.bio.ua.pt/

7 https://pubmlst.org/pputida/
