# Supplementary material for: Identification of Diverse Integron and Plasmid Structures Carrying a Novel Carbapenemase Among Pseudomonas Species
Source: Front Microbiol. 2019 Mar 4;10:404. doi: 10.3389/fmicb.2019.00404 (PMC6409357; doi:10.3389/fmicb.2019.00404)
Supplement: Supplementary file 1 [file Presentation_1.pdf]

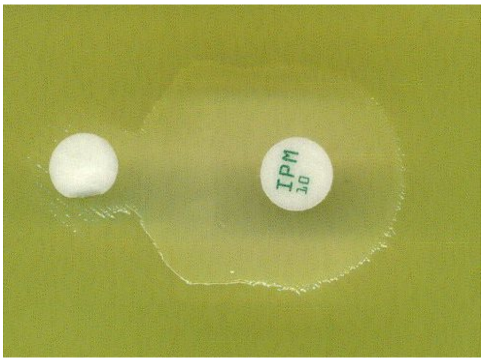

Figure S1. Double-disk synergy test with strain Pae2567. A disk loaded with EDTA (on the left) was deposited near an imipenem disk (on the right, BioRad) on a Mueller-Hinton agar medium pre-inoculated with a 0.5 McFarland suspension of strain Pae2567. The synergy zone between the two disks after 18 h incubation is indicative of production of a metallo-ß-lactamase, inhibited by zinc chelator EDTA.
